# Concussion Health Improvement Program (CHIP): study protocol for a randomized controlled optimization trial for youth with persistent post-concussive symptoms

**DOI:** 10.1186/s13063-024-08494-y

**Published:** 2024-10-09

**Authors:** Carolyn A. McCarty, Tayler Hennings, Chuan Zhou, Emily F. Law, Douglas Zatzick, Sara P. D. Chrisman

**Affiliations:** 1 Division of General Pediatrics, Department of Pediatrics, University of Washington School of Medicine, Seattle, USA; 2 Center for Child Health, Behavior, and Development, Seattle Children’s Research Institute, Seattle, USA; 3 Department of Pediatrics, and Department of Psychiatry & Behavioral Sciences, University of Kansas Medical Center, Kansas City, USA; 4 Department of Psychiatry and Behavioral Science, University of Washington School of Medicine, Seattle, USA; 5 Division of Adolescent Medicine, Department of Pediatrics, University of Washington School of Medicine, Seattle, USA

**Keywords:** Brain concussion, Cognitive behavioral therapy, Mental health, Psychosocial intervention, Case management, Randomized clinical trial, Adolescent health, Sports injury

## Abstract

**Background:**

Up to 1.9 million youth in the USA sustain a concussion each year, and up to 30% experience persistent post-concussive symptoms (PPCS) lasting 1 month or more. PPCS can substantially interfere with social, emotional, and academic functioning. Despite these concerns, few evidence-based treatments are available for youth with PPCS. We previously found effectiveness in treating youth with concussion using a collaborative care intervention that integrates mental health care into a medical model, with improvements in concussive symptoms and quality of life at 1 year. Using the multiphase optimization strategy (MOST) framework, we now aim to assess the contribution of each of the three components that were part of collaborative care: concussion-focused cognitive behavioral therapy (cf-CBT), parenting skills training (PST), and care management (CM).

**Methods:**

The MOST factorial design examines all three intervention components with two levels of each (present or absent), resulting in 8 possible treatment combinations. We will recruit 368 youth with PPCS from 2 geographic locations (Seattle and Dallas), randomizing them to 1 of 8 treatment groups. Youth and/or parents will attend treatment sessions via video conferencing software over 3 months, and complete surveys regarding primary outcomes (concussive symptoms and health-related quality of life) and secondary outcomes (sleep, pain, mood, and parental distress) at 6 weeks and 3, 6, and 12 months. We will also assess potential mediators and moderators to allow for future tailoring and refinement.

**Discussion:**

The overarching goal of this investigation is to determine which collaborative care components (delivered individually or in combination) are most effective in treating PPCS in concussion-exposed youth. The investigation will inform mental health screening, intervention, and referral procedures for youth and families following concussion. At the completion of this study, we will have an optimized and refined intervention for youth with PPCS ready for large-scale implementation and dissemination.

**Trial registration:**

ClinicalTrials.gov NCT06036147. Registered on September 13, 2023.

## Administrative information

Note: the numbers in curly brackets in this protocol refer to SPIRIT checklist item numbers. The order of the items has been modified to group similar items (see http://www.equator-network.org/reporting-guidelines/spirit-2013-statement-defining-standard-protocol-items-for-clinical-trials/).

**Table:** 

Title {1}	Concussion Health Improvement Program (CHIP): study protocol for a randomized controlled optimization trial for youth with persistent post-concussive symptoms
Trial registration {2a and 2b}.	ClinicalTrials.gov NCT06036147. Registered on September 13, 2023.
Protocol version {3}	Version 4. December 20, 2023.
Funding {4}	NICHD: RO1HD090230
Author details {5a}	1. Division of General Pediatrics, Department of Pediatrics, University of Washington School of Medicine 2. Center for Child Health, Behavior, and Development, Seattle Children’s Research Institute, Seattle, WA 3. Department of Pediatrics, and Department of Psychiatry & Behavioral Sciences, University of Kansas Medical Center 4. Department of Psychiatry and Behavioral Science, University of Washington School of Medicine 5. Division of Adolescent Medicine, Department of Pediatrics, University of Washington School of Medicine
Name and contact information for the trial sponsor {5b}	*Eunice Kennedy Shriver* National Institute of Child Health and Human Development. (NICHD). PO Box 3006, Rockville, MD 20847. 1–800-370–2943
Role of sponsor {5c}	The study funder is not playing a role in study design, data collection, analyses, or publications.

## Introduction

### Background and rationale {6a}

Youth with persistent post-concussive symptoms (PPCS) pose a rising public health challenge. Estimates suggest up to 1.9 million youth less than 18 years old sustain a concussion annually in the USA [1]. While concussion normally resolves within days to weeks following injury, up to 30% of youth experience symptoms such as headache, mood alteration, sleep disruption, and difficulty concentrating lasting longer than 1 month, currently defined as persistent post-concussive symptoms (PPCS) [2–5]. PPCS confers marked functional impairment interfering with academic performance and social interaction, and resulting in decreased quality of life [6–8]. Individuals who develop PPCS represent a small proportion of those injured, yet a disproportionate number of those requiring subspecialty care [9, 10]. Research suggests premorbid mental health issues increase the risk of developing PPCS following concussive injury [11]. However, despite recognition of the relationship between mood and PPCS, the standard of care for PPCS does not currently include mental health intervention and instead revolves primarily around rehabilitative exercise [12]. It is thus imperative that we develop effective treatments for youth with PPCS that target mental health.

Over the past two decades, our interdisciplinary investigative team has conducted a series of epidemiologic, RCT, and implementation studies that constitute foundational research for the investigation outlined in this paper [13–17]. Through this work, we have documented the association between concussion injury and a broad profile of functional impairments among youth and adults. Our initial RCT established the feasibility and acceptability of the collaborative care (CC) intervention model for injured adolescents and their caregivers, and the recent larger R01 trial demonstrated the effectiveness of this approach [14, 15].

Collaborative care utilizes principles from chronic disease management to create linkages between medical and mental health providers and has been shown to be efficacious for treating adults with mental health disorders [18, 19] occurring in the context of injury or trauma [20, 21]. In the CC approach, a care manager provides oversight and supports communication between medical and mental health teams, scaffolding individuals to navigate the medical system and improve both symptoms and overall function. We adapted this approach for youth with concussion by developing a multipronged approach to working with concussed youth and their families to address concussion symptoms, including concussion-focused cognitive behavioral therapy (cf-CBT) for youth, sessions to support parenting skills (parenting skills training, PST), and care management (CM) to scaffold coordination of concussion appointments and referrals, including medication consultation as needed [13]. We completed a series of studies examining the overall CC approach for youth, noting improvements in concussion symptoms and health-related quality of life at 12 months (Cohen’s *d* = 0.32 and 0.29, respectively), compared to usual care [14].

The current study aims to streamline the CC approach to improve efficiency and scalability, by assessing the separate contributions of three components of the intervention: (a) cf-CBT (therapy with the youth to gain knowledge and learn skills), (b) PST (parent training regarding strategies for supporting youth and strengthening relationships), and (c) CM (logistical support for families regarding navigating services such as medical and school supports).

Optimizing the CC intervention will decrease the resources required for implementation, thereby improving reach, particularly for underserved minority families or those with lower socioeconomic status. While we develop evidence for the CC approach, we also want to ensure equitable access. Prior research suggests disparities in receipt of targeted treatment post-concussion, with differences most profound for racial and ethnic minority groups and lower-resourced individuals [22, 23]. Research on psychosocial interventions with minority youth suggests that focusing on systemic barriers, including developing shorter interventions, is more effective than cultural tailoring [24]. Optimizing the CC intervention into a shorter package that is more readily deliverable to families should improve treatment completion, particularly among those with more limited resources [25, 26].

**Fig. 1:**
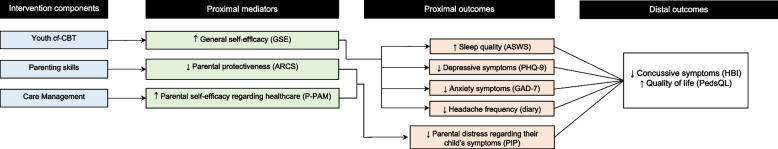
Conceptual model of the CC intervention

Understanding the mechanistic factors that mediate the benefits of the CC intervention will provide a means to further enhance effectiveness. The CC intervention has multiple components, each of which targets specific behaviors in parents and youth. We have outlined these potential mediators in our conceptual model (Fig. 1) and will test these treatment groups analytically. We anticipate youth receiving cf-CBT should report increased self-efficacy regarding managing their symptoms, and parents receiving PST should report decreased parental protectiveness. Care management is focused on helping families navigate care, and thus should result in increased self-efficacy regarding managing their child’s health care. We believe the effectiveness of each component for changing our primary outcomes will be secondary to these factors, as shown in Fig. 1, and we plan to test these hypotheses using formal mediation analysis. We have also outlined potential proximal outcomes (youth pain, sleep and mood, and parental distress regarding their child’s illness), which may underlie the relationship between mechanistic factors and distal outcomes, and we will assess these as potential secondary outcomes.

Exploring the moderation of component effects by youth and parent characteristics will allow for further refinement of the CC approach. While we work to refine and narrow the intervention, we recognize that the needs of families vary, and certain youth or family characteristics might predict greater benefits for a particular component. Characterizing treatment moderators is essential for maximizing treatment response, as there may be identifiable factors that create additional barriers to recovery. For example, in our recent R01 study, youth with a history of mental health issues were more likely to experience a severe persistent trajectory of concussive symptoms (although this was still less likely in intervention compared to control) [27]. One could theorize that such youth might require intensive youth-focused treatment to be a part of the treatment approach (i.e., cf-CBT), or need additional support along with cf-CBT (i.e., PST, CM) [27]. Families with parents who have a higher level of distress at baseline might benefit more significantly from intervention components providing skills to manage such distress (i.e., PST or CM) [28–30]. In addition, prior research regarding moderate-severe traumatic brain injury (TBI) has found differential effectiveness of parent-oriented treatments by parental education level, with parents from lower educational levels experiencing the most gain [31]. We will thus assess moderation by parental education level, hypothesizing that parents with lesser education might have greater benefit from engagement in treatment, either via the parenting skills training component or care management. We will also explore moderation by sex and race/ethnicity, even though these were not significant moderators in prior studies, with the recognition that these factors might only impact the effectiveness of one intervention component.

The CC intervention model is innovative in that it addresses mental health in youth with PPCS by supporting both youth and parents. The current trial aims to optimize and refine this approach to improve efficiency and scalability, while ensuring maximal intervention effect. Following the completion of this optimization trial, we will have data regarding the contribution of each component to treatment effects, including potential mediating and moderating factors, utilizing a diverse population from two geographic locations. We will then refine the CC intervention to include only those components that efficiently improve youth outcomes in order to implement and disseminate this intervention on a broader scale.

### Objectives {7}

Our first aim is to determine which components of the CC approach (cf-CBT, PST, and CM) contribute significantly to improvements in distal outcomes, particularly concussive symptoms, and youth health-related quality of life (HRQoL), among a diverse sample of youth with PPCS. We hypothesize that over the course of the 12-month trial, families who receive cf-CBT, PST, or CM will demonstrate greater improvements in the primary outcomes compared to those who do not receive these components, but that these components will be differentially beneficial, suggesting that a streamlined intervention may be comparably effective. As part of this aim, we plan to examine the effect of intervention components on proximal outcomes including youth headache, mood and sleep, and parental distress regarding their child’s illness.

Our second aim is to assess the potential mediation of intervention component effects by postulated mechanistic factors including improvements in (a) youth self-efficacy, (b) parental protectiveness, and (c) parental self-efficacy regarding navigating their child’s concussion care. We hypothesize that youth receiving cf-CBT will report increased self-efficacy regarding CBT; that parents receiving PST will report decreased parental protectiveness; and that families receiving care management will report higher self-efficacy regarding managing their child’s concussion care.

Our third aim is to explore the moderation of intervention component effects by demographic factors including parental education level, youth race/ethnicity, and youth sex, and clinical factors, including youth depression and level of parent emotional distress.

### Trial design {8}

Multiphase Optimization Strategy (MOST) is an innovative, engineering-inspired framework to build optimized interventions [32]. Using this framework, we will use a factorial design to assess the differential effects of the three components of the CC intervention (cf-CBT, PS, and CM) on post-concussive symptoms and health-related quality of life. Given that we have three components and two levels of each (present or absent), adolescents participating in the study will be randomized to one of 2^3^ = 8 combinations of treatment components, yielding 8 different treatment groups.

## Methods: participants, interventions, and outcomes

### Study setting {9}

Recruitment will primarily take place at two study sites, Seattle Children’s Hospital and University of Texas, Southwestern. Recruitment from other states is also permitted, but no specific recruitment efforts are underway elsewhere aside from the presence of a study website. Most recruitment will be carried out remotely via chart review and reaching out to patients via a variety of modalities. All aspects of the study intervention and data collection will be delivered remotely.

### Eligibility criteria {10}

Inclusion criteria for each subject population:

- Youth
-
  - Eleven to 18 years old
  - Health care provider diagnosed concussion (as outlined by the 6^th^ International Conference on Concussion in Sport)
  - ≥ 3 new onset or worsening post-concussive symptoms
  - One to 12 months following injury
  - Fluent in English or Spanish

- Parents
-
  - Legal guardian or parent of an eligible youth participant who enrolls in the study
  - Fluent in English or Spanish

Exclusion criteria for each subject population:

- Youth
-
  - Acute suicidal ideation (an active plan)
  - Diagnosis of psychosis
  - Psychiatric hospitalization within the past 6 months
  - Spinal cord or other severe injuries
  - Chronic illness or medical conditions that prevent participation in concussion-focused treatment

### Who will take informed consent? {26a}

Formed consent will be conducted over the phone or via video conference with one of the research coordinators for the study. Youth < 18 will provide assent and parents will provide parental permission and will also consent for themselves. Parents and youth will consent by electronically signing REDCap e-consent or assent forms. Informed consent procedures have been approved by the Seattle Children’s Institutional Review Board.

### Additional consent provisions for collection and use of participant data and biological specimens {26b}

N/A, no biological specimens will be collected.

## Interventions

### Explanation for the choice of comparators {6b}

The study includes 8 different treatment arms, as specified in Table 1 below.

**Table 1:** Intervention treatment groups

Treatment group	Percentage in this group	Sessions
1	12.5%	Treatment group 1 receives cognitive behavioral skills for concussion, parenting skills training, and care management (19 sessions total)
2	12.5%	Treatment group 2 receives cognitive behavioral skills for concussion and parenting skills training (13 sessions total)
3	12.5%	Treatment group 3 receives cognitive behavioral skills for concussion and care Management (13 sessions total)
4	12.5%	Treatment group 4 receives cognitive behavioral skills for concussion (7 sessions total)
5	12.5%	Treatment group 5 receives parenting skills training and care management (13 sessions total)
6	12.5%	Treatment group 6 receives parenting skills training (7 sessions total)
7	12.5%	Treatment group 7 receives care management (7 sessions total)
8	12.5%	Treatment group 8 receives surveys only (0 sessions)

### Intervention description {11a}

#### Introductory session

All participants assigned to treatment groups 1–7 will be invited to participate in an introductory session, which includes psychoeducation and collaborative development of goals for treatment. For treatment groups 1, 2, and 3, both youth and parents will participate in the introduction session. For treatment group 4, only the youth will participate in the introduction session. For treatment group 6, only the parent will participate in the introduction session. For treatment groups 5 and 7, the parent will participate with the youth optional.

#### Component 1: concussion-focused cognitive behavioral therapy (cf-CBT)

The cf-CBT component targets post-concussive symptoms including mood, pain, and sleep by teaching adolescents established evidence-based emotion regulation, relaxation, and behavioral and cognitive strategies to manage their symptoms. Homework/practice assignments are given to help adolescents apply in-session concepts to the management of their symptoms. Youth randomized to receive this component will participate in six 30-min sessions of cf-CBT.

#### Component 2: parenting skills training (PST)

The focus of the PST component is on the use of positive parenting skills as well as helping parents manage their own emotional distress. Parents are taught to set positive recovery expectations and to use praise or attention to increase their teen’s positive coping behaviors and decrease unhelpful coping behaviors. They are also taught positive communication skills to use with their teen, and guided to schedule pleasant events together in order to strengthen the relationship. In vivo skills practice and feedback are provided to help parents generalize and use parenting techniques. Parents randomized to receive this component will participate in six 30-min sessions of PST.

#### Component 3: care management (CM)

In the CM component, parents are provided support regarding advocating for their child’s needs across different contexts in the healthcare system, school, and athletic departments (including guidance regarding medication referrals), using applied problem-solving together with the skills coach to address emergent needs. Adolescents can also be involved in CM, if developmentally appropriate based on their age, independence, and interest. Of note, while the CM component engages youth and parents, treatment effects are hypothesized to result from facilitating access to supportive services including medication referrals at the organizational level. Families randomized to receive this component will participate in six 30-min sessions of CM.

Treatment groups 1–7 will receive 7–19 sessions based on their group assignment (see Table 1) and these will be delivered over 12 weeks. Each half-hour intervention session will only address one of the three unique components, and the cadence of delivery of each component (Table 2) will allow all sessions to be completed during the 12-week intervention time frame, with a maximum of 10 h of intervention for families receiving all components. Components will be interspersed to allow time to practice and use skills in between sessions, and to mimic the manner in which treatment would be delivered clinically.

**Table 2:** Description of intervention components and dosage

Intervention component	Primary recipient	Core intervention elements	Intervention dosage
Concussion-focused cognitive behavioral therapy (cf-CBT)	Youth	Emotion regulation, relaxation training, behavioral and cognitive strategies to manage symptoms	6 half-hour sessions, delivered within the first 3 months
Parenting skills training (PST)	Parent	Parenting behavior, stress management, and positive family communication	6 half-hour sessions, delivered within the first 3 months
Care management (CM)	Parent and youth	Care coordination coaching across medical, educational, and sports settings; psychoeducation and working with medical providers on medication referrals when needed	6 half-hour sessions, delivered within the first 3 months

#### Usual care for treatment group 8

Patients randomized to treatment group 8 will only receive the usual care that they seek from their health care provider, without additional intervention elements. Usual post-concussive care includes the routine use of sports medicine, rehabilitation medicine, primary care, and emergency department services, as well as the occasional use of other specialty providers (such as physical therapy or mental health).

### Criteria for discontinuing or modifying allocated interventions {11b}

Participants will be allowed to discontinue receiving any of the study intervention components at any time point, but would be retained for study analyses. The dosage of intervention received will be tracked for all participants.

### Strategies to improve adherence to interventions {11c}

We are using five strategies to ensure interventionist fidelity to participants’ assigned condition and minimize the risk of contamination (consistent with NIH & Template for Intervention Description and Replication (TIDieR) guidelines [33, 34] and optimization trial procedures [35]: (1) standardized interventionist training; (2) auto-generated, condition-specific charting templates generated in REDCap, so that interventionist can only document on component levels assigned to specific participants; (3) condition-specific scripts, so interventionists deliver and discuss only material assigned for that condition; (4) fidelity ratings of audio-recorded intervention sessions (~ 10%) to prevent interventionist drift; and (5) weekly interventionist meetings to review participants’ progress and intervention receipt.

### Relevant concomitant care permitted or prohibited during the trial {11d}

Regardless of group assignment, participants may also receive usual care from their health care provider. Usual post-concussive care includes the routine use of sports medicine, rehabilitation medicine, primary care, and emergency department services, as well as the occasional use of other specialty providers (such as physical therapy). Patients infrequently receive mental health care. Usual care at our study sites follows the Centers for Disease Control and Prevention’s (CDC) standards of care for pediatric concussion.

### Provisions for post-trial care {30}

Participants who ask for additional resources or who present in need (e.g., more acute mental health issues emerge) will be provided with referrals to specialty care at the end of the intervention period (e.g., 3 months after baseline).

### Outcomes {12}

#### Baseline measures

##### Prior psychiatric history

We will collect information from participants on prior psychiatric disorders based on our experience with prior studies on adolescents and adults. We will collect information on prior history of treatment for psychiatric disorders, use of psychotropic medications, and prior post-concussive symptoms [36].

##### Demographic characteristics

Parents will be asked to describe family demographic characteristics such as family configuration, race, and ethnicity.

Caregiver(s)’ occupations and combined family income will be also be obtained and used as a measure of family resources.

##### Health service and medication utilization

Parent reports will be used to assess adolescents’ pre- and post-sports injury health service utilization. Parents will report the specific number of visits to the emergency department, primary care provider, and subspecialty providers over the prior 6 months. Parents will also report on the types of medication used by adolescents (Table 3 ).

**Table 3:** Study assessments, use, and administration

Construct	Measure	How used	Baseline	6 weeks	3 months	6 months	12 months
Post-concussive symptoms	HBI	Primary outcome	A	A	A	A	A
Health-related quality of life	PedsQL	Primary outcome	A, P	A, P	A, P	A, P	A, P
Depressive symptoms	PHQ-9	Proximal outcome; moderator	A	A	A	A	A,
Anxiety symptoms	GAD-7	Proximal outcome	A	A	A	A	A
Sleep quality	ASWS-SF	Proximal outcome	A, DD	A	A, DD	A, DD	A, DD
Headache pain	NRS-11	Proximal outcome	DD		DD	DD	DD
Treatment acceptability and satisfaction	TEI-SF	Proximal outcome			A, PS		
Parent distress regarding their child’s illness	PIP	Proximal outcome; moderator	PS	PS	PS	PS	PS
Parent self-efficacy regarding engaging with health care	P-PAM	Proximal outcome, mediator	PS	PS	PS	PS	PS
Adolescent self-efficacy	GSE	Mediator	A	A	A	A	A
Parental protectiveness	ARCS	Mediator	PS	PS	PS	PS	PS
Youth prior psychiatric and concussion history		Descriptive	P				
Demographic characteristics		Descriptive	A, PS				
Health care utilization		Descriptive	P			P	P

*A* adolescent, *P* parent report of adolescent, *PS* parent self-report, *DD* daily diary

#### Primary outcome measures

Health Behavior Inventory (HBI): A 20-item questionnaire that assesses concussive symptoms on a 4-point scale, ranging from “never” to “often,” and yields total scores in cognitive and somatic domains. We will use the youth-report version with established reliability and validity in youth with sports injury [37].

Pediatric Quality of Life Inventory (PedsQL): A 23-item questionnaire that assesses physical, emotional, social, and school functioning. The scale includes youth-report and parent-report versions [38–40].

#### Secondary outcome measures

Patient Health Questionnaire (PHQ-9): A 9-item questionnaire that measures the severity of depressive symptoms. Reliability and validity of the PHQ-9 have been established in pediatric populations with injury [41].

Anxiety measures (GAD-7): A 7-item standardized anxiety measure that asks youth to rate how often they have been bothered by anxiety symptoms using a 4-point Likert scale. It has been shown to have good reliability, as well as criterion, construct, factorial, and procedural validity for assessing anxiety [42].

Adolescent Sleep Wake Scale: The 10-item version of the ASWS, including domains of falling asleep, reinitiating sleep, and returning to wakefulness will be used as a measure of sleep quality, based on adolescent report [43].

Daily diary (DD): The 7-day electronic daily diary surveys will include headache, mood, and energy level measures. To assess headache frequency, adolescents will report whether or not they had a headache each day [44] and will rate intensity using an 11-point numerical rating scale (NRS-11) ranging from 0 (“no pain”) to 10 (“worst pain ever”) [45]. These headache frequency and intensity items will be texted and/or emailed in the evening. This electronic 7-day short daily survey has been used successfully to assess headache frequency and pain intensity in prior studies of adolescents [44, 46, 47].

Treatment Evaluation Inventory Short Form (TEI-SF): A 6-item self-report measure that assesses participant perceptions of acceptability and satisfaction with the treatment program on a 5-point scale ranging from “strongly disagree” to “strongly agree.” The TEI-SF has demonstrated adequate reliability and validity across a wide array of treatment studies [48].

Pediatric Inventory for Parents (PIP): The 15-item emotional distress subscale of the PIP will be used to measure the frequency and difficulty of illness-related parenting distress [49].

#### Potential mediators

Parent-Patient Activation Measure (P-PAM): [50]. The 10-item P-PAM will be used to assess parenting self-efficacy in advocacy within the health care system (knowledge, skills, and confidence for managing their child’s health care) [51]. The 4-point scale ranges from “disagree strongly” to “agree strongly” (e.g., “I am confident I can tell a doctor concerns I have about my child’s health”).

Adult Responses to Children’s Symptoms (ARCS): The 13-item Protect Scale and 4-item Monitor Scale from the Adult Responses to Children’s Symptoms (ARCS) [52] measure will be used to assess frequencies of parental protection and monitoring behaviors, with all items rated on a Likert scale ranging from 0 (never) to 4 (always). We adapted this measure to concussion by replacing “pain” with “concussive symptoms,” and it will be given to both adolescent and parent participants.

General Self-Efficacy Scale (GSE): This 10-item scale measures general self-efficacy on a scale of 1 (not at all true) to 4 (exactly true) on items such as “I can usually handle whatever comes my way,” with higher scores indicating greater self-efficacy [53]. Youth will provide self-report on this measure.

### Participant timeline {13}

**Fig. 2:**
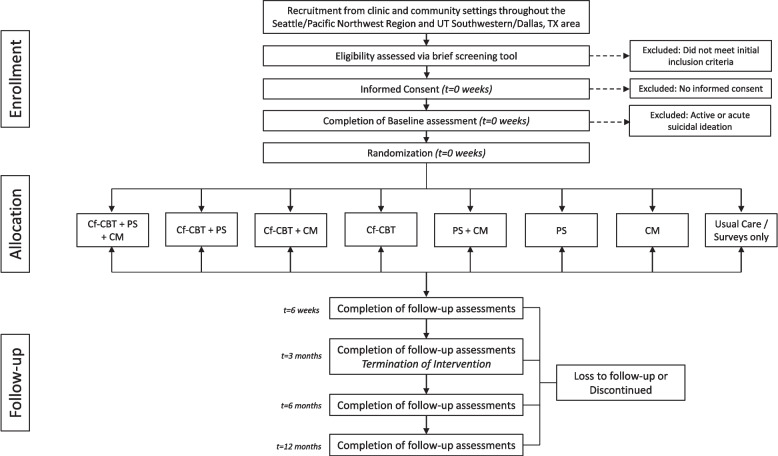
Consort trial flow diagram

The participant timeline is shown in Fig. 2.

### Sample size {14}

We plan to enroll a total of 374 youth-parent dyads across all participating study sites. Three hundred sixty-eight participants will be included in the analyses. The six other participants are considered “training participants,” who will not be randomized and will receive treatment pathway 1 (all intervention components) for the purpose of providing an opportunity for each interventionist to practice intervention delivery with 1–2 families prior to the enrollment of actual study participants. Power analyses were conducted using FactorialPowerPlan SAS Macro for Aims 1 and 3, and PASS for Aim 2 to determine the appropriate number of participants for the study. The factorial design involves 3 completely crossed factors, and thus the same power calculation applies to each factor being tested, and to interaction effects also, since this is a 2^3^ factorial. Based in part on prior investigations by the study team,^1^ assumptions include: 5 assessment points (baseline, 6 weeks, 3 months, 6 months, and 12 months), within-subject correlations over time of *ρ* = 0.6, two-tailed alpha = 0.05, and 10% 12-month attrition were used for all power analyses. In the prior single-site R01 trial, the study team observed 6–12-month HBI treatment effects ranging between 0.26 and 0.32 and PedsQL treatment effects ranging between 0.19 and 0.29. The proposed sample size of 368 (184 per factor present vs. absent comparison) provides power > 0.80 to detect a small effect size of Cohen’s *d* = 0.15 under these assumptions. In terms of the cell size of the factorial design, we will allocate 46 participants to each of the 8 groups and retain an average of 42 participants within each. In terms of power to detect mediation for Aim 2, with “a” the standardized coefficient between the component group and the mediators = 0.34, “b” the standardized coefficient between the mediators and PedsQL/HBI symptom outcomes = 0.34, and with 368 patients, 10% attrition and alpha set at 5%, the power = 0.80 to detect minimal indirect/mediated effect 0.12 [54]. With a total sample size of 368, the component group by moderator interaction effect size was estimated using ANCOVA with baseline HBI or PedsQL as the covariate. With the probability of a 2-tailed type I error set at 5%, the study will have ≥ 0.80 power to detect a component group by moderator interaction ES = 0.25 for 12-month HBI or PedsQL.

### Recruitment {15}

Recruitment will take place from several sources:

Seattle Children’s Hospital: We will recruit from Sports Medicine, Rehabilitation Medicine, Adolescent Medicine, and Neurology clinics as well as the Emergency Department/Urgent Care within Seattle Children’s Hospital. There will be active recruitment by weekly scanning of the appointment lists for these sites. Providers seeing potentially eligible subjects will be contacted prior to patient visits and asked to provide the patient with study information, including a study flyer and/or a recruitment letter with a QR code that they can scan on their phones and will lead them to the CHIP Study REDCap Screening Form. Clinicians will be provided a dot phrase to share with patients if they would like, explaining the CHIP study. If a patient did not receive study information at their visit, we will send them a recruitment letter, briefly explaining the study and options to contact us, and will then text or call them to assess interest (a maximum of 5 additional attempts). Potential participants will also be provided the link to the study website, https://chipstudy.org. Our website will be public and we are able to recruit participants from anywhere in the USA if they come across the study online and meet eligibility requirements.

University of Texas, Southwestern (Dallas, TX): We will recruit from Sports Medicine, Rehabilitation Medicine, and Neurology providers at UTSW and two other organizations associated with UTSW: Children’s Medical Center and Scottish Rite for Children. The UTSW research assistants will approach patients in person and fill out the CHIP REDCap screening form if they are interested. Seattle Children’s research will take over the recruitment process post-screening form.

#### Assignment of interventions: allocation

##### Sequence generation {16a}

Randomization will occur in blocks according to a computer-generated random assignment and stratified by patient’s sex assigned at birth (male or female) and study site (Seattle, UTSW, or other community). Once the randomization spreadsheet is generated, it will be uploaded into the REDCap randomization module.

##### Concealment mechanism {16b}

Once the randomization spreadsheet is generated by the study statistician, it will be uploaded into REDCap. No one will be able to see the allocation sequence until the “randomize” button in REDCap is clicked and the next participant is assigned to a group.

##### Implementation {16c}

Randomization will occur directly in REDCap by the study coordinator. After randomization, a letter and email will be sent to the participant’s family to notify them of which group they have been randomly assigned.

#### Assignment of interventions: blinding

##### Who will be blinded {17a}

Only the study statistician and fidelity coders will be blinded to the treatment pathways. The data will be coded with a number sequence to indicate the treatment pathways. The research coordinators will have access to the data dictionary with the codes.

##### Procedure for unblinding if needed {17b}

Unblinding of the statistician will only occur after data has been analyzed.

#### Data collection and management

##### Plans for assessment and collection of outcomes {18a}

All outcome data will be collected using self-report surveys in REDCap. Surveys will be completed at 6 weeks and 3, 6, and 12 months after enrollment and will be automatically triggered. See the “Primary outcome measures“ section for detailed descriptions of the measurements.

##### Plans to promote participant retention and complete follow-up {18b}

Research coordinators will be tracking survey completion regularly and reaching out to participants with reminders. Our participant incentive will be tied to their survey completion, to encourage participants to complete surveys on time. Additionally, we will send each of our participants a study gift at 3 months to keep them engaged in the follow-up data collection.

##### Data management {19}

Surveys, video-recorded sessions, and other data will be obtained during the study. Surveys and participant information will be collected and stored in a REDCap project. Video recordings will be stored in a secure Seattle Children’s shared drive.

##### Confidentiality {27}

All study data will be gathered strictly for research purposes and only accessed by study staff. To ensure subject confidentiality, all research surveys and other research materials will be kept in a locked file cabinet in our secure research space, and all computers used in the collection and storage of data will be password-protected or in a HIPAA-compliant database (REDCap). After data is collected, information that would identify the subjects will be removed and code numbers used instead. A study code will be assigned to each subject. Data stored in REDCap will have PHI variables indicated such that a deidentified dataset could be obtained as needed.

##### Plans for collection, laboratory evaluation, and storage of biological specimens for genetic or molecular analysis in this trial/future use {33}

N/A, this study does not collect any biologic specimens.

## Statistical methods

### Statistical methods for primary and secondary outcomes {20a}

#### Aim*** 1***

For Aim 1, the primary purpose of the statistical analyses is to determine whether the three intervention components (a) cf-CBT, (b) PST, and (c) CM each contribute significantly to improvements in post-concussive symptoms (HBI) and youth HRQoL (PedsQL). We hypothesize that over the course of the 12-month trial, families who receive cf-CBT, PS, or CM will demonstrate greater improvements in the primary outcomes compared to those who do not receive these components but that they may be differentially beneficial, suggesting that a streamlined intervention may be comparably effective. To examine this hypothesis, we will perform two sets of analyses. The first set of analyses will examine the effects of individual components (cf-CBT, PST, CM). Towards this end, we will use effect coding of variables for the three components and fit mixed regression models with the following conditional mean structure specification:

$$E\left(Y_{ij}\right|b_{0i})=\beta_0+\beta_1cfCBT_i+\beta_2PST_i+\beta_3CM_i+\beta_4Time_j+\beta_5cfCBT\times Time_j+\beta_6PST_i\times Time_j+\beta_7CM_i\times Time_j+\beta_8cfCBT_i\times PST_i+\beta_9cfCBT_iCM_i+\beta_{10}PST_i\times CM_i+other\;interactions+\gamma X_i+b_{0i}$$

where $cfCBT_i\;=\;1$  if patient $i$ received cfCBT, 0 otherwise, same for PST and CM. The*ot*ℎ*er interactions* include all other two-, three-, and four-way (3 components and time) interaction terms. Based on the mixed models, we can perform repeated ANOVA and test the main effects of each of the three components on our primary outcomes. We will also test for interactions between components.

The second set of analyses will examine the differences across the combinations of the three intervention components as a whole, and identify better-performing combination(s). For this analysis, regardless of linear or generalized linear models, the mean structure for mixed effects regression models can always be specified as

$$(Y_{ij})\;=\;\beta_0\;+\;\beta_1CHIP_i\;+\;\beta_2Time_j\;+\;\beta_3CHIP_i\;\times\;Time_j\;+\gamma X_i\;+b_{0i}$$

where *Y*_*ij*_ is the outcome for subject *i* at time *j*, *b*_0*i*_ is the subject-specific random intercept to account for within-subject correlation due to repeated measures, and *CHIP*_*i*_ × *Time*_*j*_ is the intervention-by-time interaction term. *CHIP*_*i*_ takes value 1–8 representing each of the 8 combinations of cf-CBT, PS, and CM. *X*_*i*_ is a vector of covariates including baseline injury severity, birth sex, and referring site. We will model *Time*_*j*_ as a discrete variable given a limited number of assessments, the coefficients of the intervention-by-time interaction β_3_ estimate the mean differences between treatment arms in changes (“difference-in-differences”) at later time points relative to baseline. The significance of these coefficients will be assessed using confidence intervals, and *p*-values from *F*-tests based on ANOVA and the Kenward-Roger method [55]. For Aim 1a, we will examine component effects on youth headache, depressive symptoms (PHQ-9) and sleep (ASWS), and parental distress regarding their child’s illness (PIP) using the same methods.

Using our importance criteria, for intervention optimization, we will consider any components or interactions demonstrating an effect size of 0.20 on either the HBI or PedsQL to be worth retaining in the optimized intervention. We will make our initial decision at 6 months, but will also examine 12-month HBI and PedsQL outcomes. We are balancing effectiveness and efficiency in our optimized intervention by not including components that demonstrate a small effect (Cohen’s *d* = 0.20) at 6 or 12 months on the HBI or PedsQL.

#### Aim 2

Mediational analyses will account for the temporal associations between treatment group assignment, hypothesized mediators, and the PedsQL and HBI outcomes at 6 months. For the mediational analyses, the component assignment will be the predictor variable. For these analyses, component comparisons of the mediators (youth self-efficacy regarding CBT, parental protectiveness, and parent self-efficacy regarding engaging with health care) will be assessed over the course of the first 6 months post-randomization. We hypothesize that youth receiving cf-CBT will report increased self-efficacy regarding CBT; that parents receiving PST will report decreased parental protectiveness; and that families receiving care management will report higher self-efficacy regarding managing their child’s medical care. The total intervention effect on study outcomes will be parsed into direct and indirect pathways. The study team will fit separate mediational models for the proposed mediators and will use linear regression models for the analyses of mediated data. The regression equations can be schematically depicted as follows, HBI ~ cf-CBT + Covariates,

$$\mathrm{Youth}\;\mathrm{Self}-\mathrm{efficacy}\sim\mathrm{cf}-\mathrm{CBT}+\mathrm{Covariates},$$

$$\mathrm{HBI}\;\sim\;\mathrm{cf}-\mathrm{CBT}\;+\;\mathrm{Youth}\;\mathrm{Self}-\mathrm{efficacy}\;+\mathrm{Covariates}$$

Note the first two equations correspond to cross-sectional analysis for primary and secondary endpoints. The third equation allows us to examine the association between concussion symptoms and self-efficacy in the presence or absence of a cf-CBT program.

Given these model objects, the estimation will proceed by simulating the model parameters based on their approximate asymptotic distribution (i.e., the multivariate normal distribution with the mean equal to the parameter estimates and the variance equal to the asymptotic variance estimate), and then computing causal mediation effects (average causal mediation effect, ACME) of interest for each parameter drawn. This procedure has been implemented in the R {mediation} package [56, 57]. The role of other hypothesized mediators can be assessed using the same approach.

#### Aim 3

Moderator analyses will assess whether each component impacts primary outcomes differentially based on demographic and clinical factors measured at baseline. For demographic factors, we will examine the variables of parental education (4-year college degree vs. less education); youth assigned sex at birth (male or female), and youth race/ethnicity (Hispanic, non-Hispanic white, and non-Hispanic non-white) as moderators. For clinical factors, we will examine youth depression at baseline (PHQ-9 score ≥ 10 vs. < 10) and parent distress (as measured by the PIP, dichotomized based on a median split). To test these exploratory moderators, the study team will first examine the two-way interaction of component groups by each factor measured at baseline. If a significant interaction is detected, the study team will conduct a subgroup analysis and examine each of the component effects using mixed model regression within the subgroup defined by the moderators.

### Interim analyses {21b}

No interim analyses are planned.

### Methods for additional analyses (e.g., subgroup analyses) {20b}

If a significant interaction is detected between demographic variables, youth depression, and/or parent distress, the study team will conduct subgroup analysis and examine each of the component effects using mixed model regression within subgroups for that moderator.

### Methods in analysis to handle protocol non-adherence and any statistical methods to handle missing data {20c}

The primary intention-to-treat (ITT) analyses will be based on all available data. Given the longitudinal design, we anticipate missing data will mostly occur in the outcome variables. For patient-reported outcomes, subjects may miss one or more of the 5 assessments. Repeated measures analysis based on mixed effects regression models can be performed on data with intermittent missing, that is, subjects will be included in the analysis as long as they have at least one observed outcome value.

The amount of missing data and pattern of missing data will be examined at each time point (T1–T5). Simple imputation using the “best” and “worst” scenarios for missing data will help identify the influence of missing data on the study results. If a substantial amount of missing data exists, in addition to our primary data analysis using all available data, we will conduct sensitivity analysis using multiple imputations with multivariate imputation using the chained equations (MICE) technique, assuming missing at random. Missing values are imputed based on the observed values for a given individual and the relations observed in the data from other participants, assuming the observed variables are included in the imputation model. Multiple imputation procedures, particularly MICE, are very flexible and can be used in a broad range of settings. Because multiple imputation involves creating multiple predictions for each missing value, the analyses of multiply imputed data take into account the uncertainty in the imputations and yield more accurate standard errors. The MICE approach is a flexible yet powerful technique to address missing data issues and has demonstrated excellent performance in practice [58–60].

### Plans to give access to the full protocol, participant-level data, and statistical code {31c}

The PIs and co-investigators are willing to share data and materials with other eligible investigators through academically established means as soon as they have published their research results. We accept full responsibility to protect the rights of participants and the confidentiality of the data collected in this study. In these data-sharing efforts and with any others that may arise in the future, we will enter into data-sharing agreements prior to exchanging study data. These agreements will include the following requirements (1) researchers outside of the study team will submit a research plan proposal for PI approval prior to receipt of any data, (2) data recipients agree not to attempt to identify individuals who are subjects of the data, (3) data recipients will participate in regular check-in meetings with the study PIs during the analysis process, and (4) at least one of the study PIs (Dr. McCarty or Chrisman) will review all manuscripts prior to submission to verify that data are accurately described and represented.

### Oversight and monitoring

#### Composition of the coordinating center and trial steering committee {5d}

This study does not have a coordinating center or a trial steering committee. Seattle Children’s Research Institute is the main study site with UTSW as a participating site. The study team is made up of four investigators who provide weekly oversight over study procedures, two research coordinators responsible for recruitment, informed consent, and data management, as well as three therapists who deliver the intervention components. We also have two individuals supporting data analysis, a statistician and a multiphase optimization strategy (MOST) expert, who will be particularly engaged in the later years of the trial.

#### Composition of the data monitoring committee, its role and reporting structure {21a}

The study has an external, independent DSMB. The board consists of a pediatric neuropsychologist who specializes in the assessment and treatment of youth with concussion (who will also serve as the Board Chair), a quantitative biostatistician, and an adolescent health bioethicist. The study team will meet with the DSMB approximately every 6 months throughout the study to review recruitment and follow-up, protocol deviations, and adverse events. The board will conduct reviews of study progression and data integrity, including assessments of attainment of study recruitment milestones, adequacy of follow-up, and potential sources of bias and threats to internal validity The board reports directly to the study sponsor, NICHD.

#### Adverse event reporting and harms {22}

The adolescent study population is known to experience a number of adverse events (AE) after concussion injury that are not expected to be study-related. For instance, based on prior study team investigation, it is anticipated that a subgroup of adolescents recruited into the investigation may have pre-concussion mental health symptoms that may contribute to suicidal ideation and even suicide attempts while enrolled in the investigation. Adolescent suicide attempts are defined as a serious adverse event (SAE) for the study as are any emergency department visits/hospitalizations that are associated with suicidal ideation or intent.

The study team may become aware of AEs/SAEs through routine interactions with study subjects, such as while scheduling, during baseline or follow-up surveys, or during intervention sessions Study team members will ensure that the DSMB members are made aware of any relevant problems, or SAEs.

#### Frequency and plans for auditing trial conduct {23}

The study investigators and staff meet weekly to review recruitment, retention, and intervention dosage. We have also engaged a data safety monitoring board (DSMB) that will meet annually to review recruitment progress and potential adverse events. Seattle Children’s IRB will review all study modifications and conduct an annual review of the study.

#### Plans for communicating important protocol amendments to relevant parties (e.g., trial participants, ethical committees) {25}

Any protocol modifications will be submitted to and approved by the Seattle Children’s IRB. If relevant, trial participants will be notified and ClinicalTrials.gov will be updated.

#### Dissemination plans {31a}

Results from the proposed trial will be disseminated widely through traditional pathways to the scientific community, first through conference presentations targeting academicians, primary care providers, and concussion specialists, and then via peer-reviewed publications in journals. Dissemination to community partners will occur through local and national presentations. Participants (youth and caregivers) will receive a summary of overall study results when the study concludes if they indicated interest during enrollment.

## Discussion

The overarching goal of the Concussion Health Improvement Program (CHIP) Study is to advance an understanding of which therapeutic components delivered individually or in combination are most effective in treating PPCS in concussion-exposed youth. Evidence-based treatments for youth with PPCS are urgently needed to prevent detrimental long-term sequelae.

Multiple investigations have established the effectiveness of collaborative care in reducing depressive, anxiety, and pain-related symptom presentations in adult and pediatric primary care settings [19, 61–67]. After acute injury in adults, stepped collaborative care interventions have proven their effectiveness in reducing the symptoms of PTSD and related comorbidities [21, 68, 69].

We have previously demonstrated the collaborative care approach is an effective treatment for adolescent PPCS, with long-term improvements in concussive symptoms and health-related quality-of-life compared to usual care. We are now working to optimize and refine this intervention, in order to increase the likelihood of successful implementation and dissemination. We are also seeking to broaden our research sample, recruiting from two locations (Seattle and UTSW/Dallas) which are disparate both geographically and in racial and ethnic makeup, in order to ensure generalization of study findings. The proposed two-site study using the MOST framework is a logical extension of our prior research using the CC approach, and it will result in a streamlined intervention containing only effective components, thereby improving the likelihood of uptake to enhance care in real-world clinical settings.

This study will assess a broad array of mediators in youth and parents, as delineating the pathways that drive the intervention effects and understanding the relative contribution of each will allow us to more precisely tailor and amplify the final intervention for maximum impact. We expect the benefit of the intervention flows from increased self-efficacy and decreased parental protectiveness, but confirming this will allow us to magnify treatment effects. We also plan to explore proximal outcomes including youth headache, mood and sleep, and parental distress regarding their child’s illness, in addition to the primary outcomes of concussive symptoms and health-related quality of life.

Given a goal of developing an intervention approach that works across a broad population of youth with PPCS, we feel it is essential to explore potential moderators of intervention effectiveness, including demographic characteristics such as sex, race/ethnicity, and parental education level, and also severity of pre-treatment emotional distress in parent and youth. Utilizing MOST will allow us to explore moderation by each component, and thus determine whether the package of components needs to be tailored depending on these features. Understanding how the intervention components function in different contexts and family structures will be essential for implementing this approach on a broader scale and will add to the science in this area, potentially expanding the field to new treatment approaches.

At the completion of this study, we will have a completely optimized and refined intervention for youth with PPCS ready for large-scale implementation and dissemination. The study results will inform mental health screening, intervention, and referral procedures for youth and families following concussion.

### Trial status

This trial began recruitment of participants in November 2023, and we expect to recruit the full sample near January 2027. The 12-month follow-up assessments would then be completed by March 2028. Protocol version 4; protocol version date (December 20, 2023).

## Data Availability

De-identified data will be provided to researchers outside the study team after primary analyses are completed, with PI approval.

## Abbreviations

AE: Adverse event
ANCOVA: Analysis of variance
ARCS: Adult Responses to Children’s Symptoms
ASWS: Adolescent Sleep Wake Scale
CC: Collaborative care
CDC: Centers for Disease Control and Prevention
Cf-CBT: Concussion-focused cognitive behavioral therapy
CHIP: Concussion Health Improvement Program
CM: Care management
DD: Daily diary
DSMB: Data Safety Monitoring Board
ED: Emergency department
GAD7: Generalized Anxiety Disorder-7 item
GSE: General Self-Efficacy Scale
HBI: Health Behavior Inventory
HIPAA: Health Insurance Portability and Accountability Act
HRQoL: Health-related quality of life
IRB: Institutional Review Board
ITT: Intention-to-treat
MICE: Multivariate imputation using chained equations
MOST: Multiphase Optimization Strategy
NICHD: *Eunice Kennedy Shriver* National Institute of Child Health and Human Development
NIH: National Institutes of Health
PASS: Power Analysis and Sample Size
PedsQL: Pediatric Quality of Life Inventory
PHQ-9: Patient Health Questionnaire-9 item
PIP: Pediatric Inventory for Patients
PIs: Principal investigators
P-PAM: Parent-Patient Activation Measure
PPCS: Persistent post-concussive symptoms
PST: Parenting skills training
RCT: Randomized controlled trial
REDCap: Research Electronic Data Capture
SAE: Serious adverse event
SAS: Statistical Analysis System
TBI: Traumatic brain injury
TEI-SF: Treatment Evaluation Inventory Short Form
TIDieR: Template for Intervention Description and Replication
UTSW: University of Texas, Southwestern
